# Time trends in the burden of non-COVID-19 lower respiratory tract infections among children aged 0 to 14 years

**DOI:** 10.3389/fcimb.2025.1582159

**Published:** 2025-09-22

**Authors:** Weihong Lu, Xixia Guo, Yishuai Ren, Li Wang, Tingting Xu, Yali Xu, Duoduo Li, Nali Cai, Shujun Li, Xingliang Zhang, Haibin Li, Xiangtao Wu

**Affiliations:** ^1^ Department of Pediatrics, The First Affiliated Hospital of Xinxiang Medical University, Xinxiang, Henan, China; ^2^ Department of Pediatrics, Guangdong Medical University, Zhanjiang, Guangdong, China; ^3^ Pneumology Department, Shenzhen Children’s Hospital, Shenzhen, Guangdong, China; ^4^ School of Public Health, Xinxiang Medical University, Xinxiang, Henan, China

**Keywords:** lower respiratory tract infections, children, global burden of disease, mortality, incidence, pathogens, risk factors

## Abstract

**Objective:**

This study aims to analyze the global burden, temporal trends, and main pathogenic characteristics of pediatric lower respiratory tract infections(LRTIs) across different age groups.

**Methods:**

This repeated cross-sectional study included children with LRTIs aged 0–14 years from 204 countries and regions from 1990 to 2021. The primary outcomes were cases and rates of incidence, disability-adjusted life years (DALYs), mortality, and their trends associated with LRTIs. Estimates were generated using the DisMod-MR 2.1 tool.

**Results:**

In 2021, neonates had the highest incidence and mortality. From 1990 to 2021, the global mortality rate of LRTIs in children decreased by 76.8%, with the reduction primarily driven by a 79.1% decrease in children aged 2–4 years. However, in low-middle SDI regions, the mortality rate remained as high as 4502.003 per 100,000. The primary pathogens contributing to LRTI-related DALYs and deaths in most age groups were *Streptococcus pneumoniae, Staphylococcus aureus*, and *Klebsiella pneumoniae*, while among newborns, the leading pathogens were *Klebsiella pneumoniae, Group B Streptococcus*, and *Acinetobacter baumannii.* BAPC predicted a slight improvement in the mortality rates from 18 LRTI pathogens over the next decade; however, influenza may cause an increase in childhood mortality reaching 44,820 deaths per 100,000 by 2031.

**Conclusions:**

The burden of LRTIs remains significant in low- and middle-income countries, as well as among neonates and females. While the burden of various pathogens is gradually declining, influenza warrants particular attention. Expanding vaccine coverage, improving sanitary conditions, and early interventions for high-risk children are crucial strategies to reduce LRTI burden.

## Introduction

Lower respiratory tract infections (LRTIs) are a leading cause of morbidity and mortality among children globally, particularly those under the age of 5 (Infections GBDLR and Antimicrobial Resistance C, 2024). Despite advances in medical technology and public health improvements, the global burden of LRTIs remains substantial, particularly in low- and middle-income countries. These infections, primarily caused by bacteria, viruses, or other pathogens, often manifest as pneumonia or bronchitis, significantly impacting the health and survival of children (Infections GBDLR and Antimicrobial Resistance C, 2024). The 2021 Global Burden of Disease (GBD) study highlights that, while incidence and mortality rates have declined, LRTIs still exert a profound public health burden (Safiri et al., 2022).

From 1990 to 2019, global LRTI incidence and mortality rates decreased significantly, driven largely by advancements in high-income countries. However, in low- and middle-income regions, pediatric LRTIs continue to present alarmingly high rates, influenced by factors such as air pollution, malnutrition, and inadequate sanitation (Mrcela et al., 2022; Yigezu et al., 2023). In Sub-Saharan Africa and South Asia, LRTIs remain a leading cause of child mortality (Jia et al., 2022). Studies have shown that air pollution, particularly from the use of household solid fuels for cooking and heating, contributes significantly to the high incidence and mortality rates of LRTIs (Ruan et al., 2021; Shi et al., 2023). In the coming decades, trends in pediatric LRTIs may be further shaped by increasing antimicrobial resistance, shifts in vaccine coverage, and the effects of climate change (Antimicrobial Resistance, 2022). These ongoing challenges underscore the need for a comprehensive understanding of the global burden and temporal trends of pediatric LRTIs.

*Streptococcus pneumoniae* and respiratory syncytial virus (RSV) are the most common pathogens causing LRTIs in children under 5 years old. Before the COVID-19 pandemic, influenza viruses also played an important role (Safiri et al., 2022; Wu et al., 2023). Studies have shown that the rate of RSV infection was significantly higher in low- and middle-income countries than in other regions (Wang et al., 2021). Globally, RSV-related acute LRTIs cause over 13,000 deaths annually among children under 5 years old, with the disease burden being particularly significant in Africa and Asia (Sah et al., 2023). However, an in-depth understanding of the specific distribution and impact of these pathogens on pediatric LRTIs remains lacking at different age groups.

Therefore, the limited understanding of the LRTIs risk factors among children across different regions and age groups, especially neonates and infants, hinders the development of clinical intervention strategies and global public health policies. Therefore, further research on the pathogen distribution, incidence, and mortality burden of pediatric LRTIs can greatly contribute to further understanding of the long-term impact of the pandemic and optimization of intervention measures (Zhu et al., 2022).

The 2021 GBD study represents the first update following the COVID-19 pandemic, offering new insights into the burden of LRTIs in children under 5 years old. In this study, we aim to provide a detailed analysis of the global trends, pathogen distribution, and risk factors influencing pediatric LRTIs. We also assess the indirect effects of the COVID-19 pandemic, including the “immunity debt” phenomenon. Using the updated GBD 2021 data, we conducted a subgroup analysis based on geographical regions, social development indices (SDI), age groups, and gender. Furthermore, we employed the Bayesian Age-Period-Cohort (BAPC) model to predict future trends in the epidemiology of LRTIs caused by various pathogens.

## Methods

### Study population and data collection

This study was based on data from the estimates of the 2021 GBD study (https://vizhub.healthdata.org/gbd-results/), which aimed to systematically analyze the incidence and mortality rates of non-COVID-19 LRTIs among children under 14 years old at the global, regional, and national levels from 1990 to 2021. This study included children aged 0–14 years from 204 countries and regions worldwide. In the GBD study, LRTI are defined as pneumonia or bronchiolitis (Infections GBDLR and Antimicrobial Resistance C, 2024), corresponding to International Classification of Diseases (Shin et al., 2023).

The GBD project collected data through global and regional public health organizations; hospital admission, discharge, death, and health examination records; global epidemiological surveys; and other channels. These data included the incidence, prevalence, disability-adjusted life years (DALYs) and mortality rates of various diseases, as well as health-related influencing factors. To ensure data accuracy and consistency, the GBD implemented data validation and adjustment processes. Evaluation of the incidence and mortality trends of LRTIs on a global scale was made possible by aggregating LRTI data from different regions and countries.

### Analytical methods

The GBD project used DisMod-MR 2.1, which is a Bayesian large-scale iterative hierarchical regression tool, to generate internally consistent estimates of incidence and prevalence rates. Based on estimates of DALYs and mortality rates of LRTIs, multiple stratified analyses based on age, sex, and region were performed to assess the role of different pathogens in the LRTI burden.

Analysis of Major Pathogens: The burden of multiple pathogens, including *S. pneumoniae*, *Staphylococcus aureus*, RSV, *H. influenzae*, and *Klebsiella pneumoniae*, among children in different age groups was evaluated. Determining the mortality rates associated with these pathogens across various age groups and the impact of each pathogen in different countries and regions can aid in the identification of key intervention targets.

Risk Factor Analysis: The extent and impact of 13 major LRTI risk factors, including child malnutrition, particulate matter pollution, and household solid fuel pollution, were systematically evaluated. The changes in these risk factors were assessed using historical data from 1990 to 2021.

Temporal Trend Analysis: Using historical data from 1990 to 2021, the temporal changes in LRTI burden before and after the pandemic were evaluated, taking into consideration the confounding effects of COVID-19.

Group Comparisons: Data were compared among patient groups of income level, sex, and geographic region. The study population was divided into different age groups, as follows: neonates (0–28 days), 1–5 months, 6–11 months, 1–4 years, 5–9 years, and 10–14 years. Based on the Socio-Demographic Index (SDI), 204 countries and regions are classified into different development categories: high SDI regions (0.858 < SDI ≤ 1), high-middle SDI regions(0.812 < SDI ≤ 0.858), middle SDI regions (0.670 < SDI ≤ 0.812), low-middle SDI regions (0.570 < SDI ≤ 0.670), and low SDI regions(0 < SDI ≤ 0.570) (Jin et al., 2025).

### Calculation of annual percentage changes

This study utilizes the Age-Period-Cohort (APC) model framework to analyze trends in mortality due to LRTI by age, period, and birth cohort. The APC model helps clarify the effects of age-related biological factors as well as technological and social factors on disease trends, offering advantages over traditional epidemiological analyses. It has been successfully applied to chronic diseases like cardiovascular diseases. The model fits a log-linear Poisson regression to the Lexis diagram of observed rates, quantifying the additive effects of age, period, and cohort. Given the linear relationship between age, period, and cohort (birth cohort = period − age), this study uses a Poisson-based APC model to explore LRI mortality trends. The model provides a unique perspective on how these factors interact, offering a deeper understanding of the dynamic trends in LRI mortality. For the data from 1992 to 2021, Poisson log-linear models are used to quantify and analyze LRI mortality trends.

The trends are assessed through the Estimated Annual Percentage Change (EAPC) for mortality rates and their corresponding Age-Standardized Rates (ASR). EAPC was calculated utilizing a regression model based on the natural logarithm of the ratio, expressed as: y = α + βx + ϵ.The EAPC is calculated using the formula: 100 × (exp(β) − 1), where x represents the calendar year and y is the natural logarithm (ln) of ASR. The 95% Confidence Interval (CI) for the EAPC is also estimated. If the EAPC and its lower bound of the 95% UI are both greater than 0, it indicates an upward trend in ASR; if both the EAPC and its upper bound are less than 0, it suggests a downward trend in ASR. If the EAPC crosses 0, the ASR is considered stable.

In the Bayesian Age-Period-Cohort (BAPC) model, prior distributions are assigned to the model parameters (age, period, and cohort effects) based on existing literature or expert knowledge. The likelihood of the observed data is modeled using a Poisson distribution, suitable for incidence rates. The Markov Chain Monte Carlo (MCMC) method is used to estimate posterior distributions for the parameters, providing the most probable values based on both the data and prior information. The model is then used to predict LRTI incidence rates for 2022-2031 by projecting the estimated effects of age, period, and cohort into the future, incorporating uncertainties through credible intervals (CI). After fitting the model to historical data from 1990 to 2021, forecasts for LRI incidence are generated by extrapolating the trends. The model adjusts for future changes in healthcare, environmental conditions, and other factors, reflecting the potential impacts of policy changes, medical advancements, and socio-economic improvements. Uncertainty in the forecasts is captured by the credible intervals, providing a range of possible outcomes and reflecting the inherent uncertainty in long-term predictions.

### Reporting standards

This study adheres to the relevant reporting standards for epidemiological research, particularly those concerning the analysis of temporal trends in disease burden. The data and methods used are consistent with the Strengthening the Reporting of Observational Studies in Epidemiology (STROBE) guidelines. Efforts were made to minimize bias in data collection, analysis, and interpretation. Any limitations inherent in the dataset or methodology, such as potential underreporting or misclassification of disease, are clearly stated. All rates are presented per 100,000 person-years, with 95% uncertainty intervals (UI) reported for all estimates. Uncertainty is propagated throughout each step of the burden estimation process, with the CI representing the 2.5th and 97.5th percentiles from 1,000 simulations at each stage.

By comprehensively analyzing GBD data and using the DisMod-MR 2.1 tool, this study generated global, regional, and national estimates of the incidence and mortality rates of LRTIs among children under 14 years old. The significance level was set at α = 0.05 for statistical purposes. R software (version 4.2.0) was employed for all analyses and data processing.

## Results

### Incidence of lower respiratory tract infections in children aged 0–14 years in 2021

In 2021, 69.9 million LRTI cases occurred globally among children aged 0–14 years, with an incidence rate of 3,474.42 per 100,000 (95% UI: 3,088.43–3,965.51). Among the regions stratified by the Sociodemographic Index (SDI), the low–middle region had the highest incidence and number of LRTI cases at 26.1 million. Among the countries stratified by the World Bank income classification, low–middle-income countries had the highest number of LRTI cases at 45.78 million and incidence rate at 4,519.835 per 100,000 population (95% UI 4,014.096 to 5,154.413) for children aged 0–14 years. Among the GBD super regions, South Asia had the highest number of LRTI cases at 29.89 million and incidence rate at 5,996.088 per 100,000 population (95% UI 5,191.431 to 6,777.824) (**Table 1**).

**Table 1:** Number of cases, incidence rate, and annual percentage change in incidence rate of lower respiratory tract infections in children aged 0–14 years from 1990 to 2021.

Category	Subgroup	Cases (millions)	Incidence per 100,000 (95% UI)	% change since 1990 (95% UI)
Global	All ages	69.9	3,474.42 (3,088.43-3,965.51)	-221.9% (-199.5 to -244.3)
By SDI Region	Low-middle	26.1	4,502.003 (4,004.209-5,137.527)	-195.7% (-178.2 to -213.5)
	High	5.3	1,240.15 (1,102.84-1,410.26)	-312.4% (-288.1 to -337.2)
By Age Group	Neonates (0-28d)	/	14,740.851 (13,118.822-16,546.953)	-183.2% (-162.4 to -204.7)
	1-5 months	/	12,487.260 (11,243.756-13,810.984)	-201.5% (-180.3 to -222.9)
	10-14 years	/	1,840.776 (1,445.697-2,329.717)	-68.4% (-61.5 to -75.4)
High-Burden Countries	Pakistan	/	7,531.439 (6,669.170-8,627.229)	-158.9% (-142.1 to -176.2)
	Netherlands	/	227.885 (188.185-273.606)	-401.2% (-375.8 to -427.1)

Values in parentheses are 95% uncertainty intervals. Count data are presented to three significant figures.

GBD, Global Burden of Diseases, Injuries, and Risk Factors Study; SDI, Sociodemographic Index.

Across 204 countries or regions in 2021, the incidence rate of LRTIs among children aged 0–14 years ranged from 227.885 per 100,000 population (95% UI 188.185 to 273.606) in the Netherlands to 7,531.439 per 100,000 population (95% UI 6,669.170 to 8,627.229) in Pakistan. Among children aged 0–14 years, the incidence rate was highest in newborns, at 14,740.851 per 100,000 population (95% UI 13,118.822 to 16,546.953) (**Table 1**), followed by children aged 1–5 months, with an incidence rate of 12,487.260 per 100,000 population (95% UI 11,243.756 to 13,810.984) (**Table 1**). The incidence rate decreased gradually with age, reaching 1,840.776 per 100,000 population (95% UI 1,445.697 to 2,329.717) among children aged 10–14 years (**Table 1**).

Since 1990, the global incidence of lower respiratory tract infections (LRTI) in children of all ages has decreased by 221.9% (95% UI: 199.5–244.3). When classified by SDI (Socio-Demographic Index) region, the incidence in children from low-middle SDI regions decreased by 195.7% (95% UI -178.2 to -213.5), while in high SDI regions, the decrease was 312.4% (95% UI -288.1 to -337.2). When categorized by age group, the incidence of LRTI in neonates (0–28 days) decreased by 183.2% (95% UI-162.4 to -204.7), in infants aged 1–5 months, it decreased by 201.5% (95% UI -180.3 to -222.9), and in children aged 10–14 years, the decrease was 68.4% (95% UI -61.5 to -75.4). For high-burden countries, the incidence in Pakistan decreased by 158.9% (95% UI -142.1 to -176.2), while in the Netherlands, it decreased by 401.2% (95% UI -375.8 to -427.1) (**Table 1**).

### Mortality of lower respiratory tract infections in children aged 0–14 years in 2021

In 2019, before the COVID-19 pandemic, an estimated 745,080 (95% UI 624,870 to 871,680) children aged 0–14 years died because of LRTIs, with a mortality rate of 37.144 per 100,000 population (95% UI 31.151 to 43.455). Since 1990, the mortality rate per 100,000 population has increased by 76.8%, reaching 27.123 (95% UI: 22.238–32.570). Among children aged 0–14 years, the LRTI mortality rate in neonates (0–28 days) increased by 71.5%, with a rate of 1,560.637 (95% UI 1,308.891–1,835.706), while for children aged 10–14 years, the mortality rate increased to 2.492 (95% UI 2.183–2.774) (**Table 2**).

**Table 2:** Number of deaths, death rates, and annual percentage changes in death rate of lower respiratory infections in children aged 0–14 years in 1990, 2019, 2020, and 2021.

Stratification	Group	Deaths (thousands)	Mortality per 100,000 (95% UI)	% reduction since 1990
Global	Total	550.0	27.123 (22.238-32.570)	76.8% (72.2-80.5)
By SDI	Low-middle SDI	300.0	4,502.003 (4,004.209-5,137.527)	63.1% (58.7-67.4)
	High SDI	8.2	0.891 (0.752-1.042)	92.7% (90.1-94.8)
By Age	Neonates (0-28d)	156.1	1,560.637 (1,308.891-1,835.706)	71.5% (66.8-75.7)
	10-14 years	2.5	2.492 (2.183-2.774)	47.3% (40.6-52.8)
Pathogen Leaders (2021)	*S. pneumoniae*	153.9	7.651 (6.086-9.194)	/
	*S. aureus*	54.0	2.684 (2.163-3.247)	/

Values in parentheses are 95% uncertainty intervals. Count data are presented to three significant figures.

GBD, Global Burden of Diseases, Injuries, and Risk Factors Study; SDI, Sociodemographic Index.

In 2021, there were 550 deaths due to LRTIs globally among children aged 0–14 years, with a mortality rate of 27.123 per 100,000 population (95% UI 22.238 to 32.570). Among the SDI regions, the low–middle SDI region had the highest number of LRTI deaths at 300 and mortality rate at 4,502.003 per 100,000 population (95% UI 4,004.209 to 5,137.527). Among income groups, the mortality rate in low-income countries increased by 63.1% since 1990, reaching 4,502.003 (95% UI: 4,004.209–5,137.527), while in high SDI regions, the increase was only 0.891 (95% UI: 0.752–1.042), despite a 92.7% rise. The highest number of LRTI deaths was attributed to Streptococcus pneumoniae, with 153.9 deaths and a mortality rate of 7.651 per 100,000 population (95% UI: 6.086–9.194). In contrast, Staphylococcus aureus accounted for 54.0 deaths, with a mortality rate of 2.684 per 100,000 population (95% UI: 2.163–3.247) (**Table 2**).

**Figure 1:**
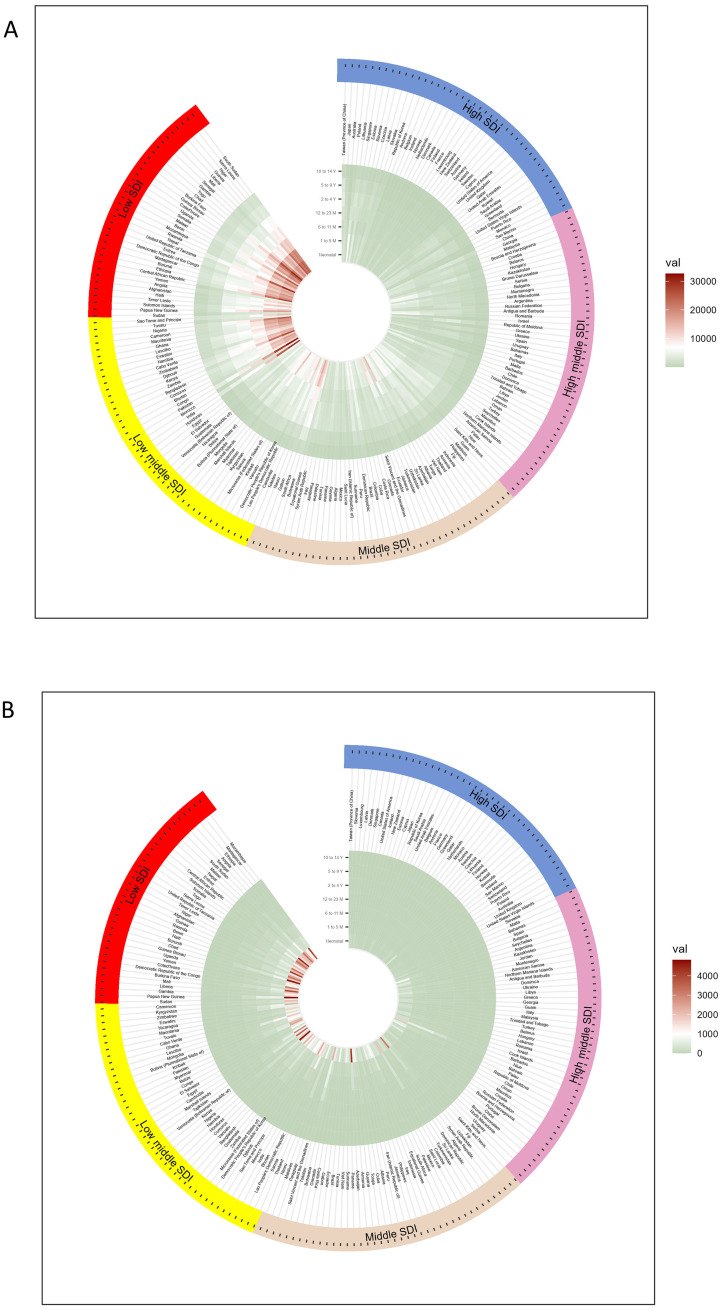
Circular heat map of the incidence and mortality of lower respiratory tract infections in children aged 0–14 years from countries with different SDI levels in 2021 **(A)** incidence and **(B)** mortality rates per 100,000 population, with colors representing global sextiles.

Across 204 countries or regions, the LRTI mortality rate among children aged 0–14 years in 2021 ranged from 151.794 per 100,000 population (95% UI 112.772 to 195.504) in Chad to 0.142 per 100,000 population (95% UI 0.097 to 0.194) in Andorra (**Figure 1**). The highest mortality rate was observed in newborns (1,560.637 per 100,000 population; 95% UI 1,308.891 to 1,835.706). In children aged 10–14 years, mortality rate decreased with age, reaching 2.492 per 100,000 population (95% UI 2.183 to 2.774) (**Table 2**).

In 2021, the number of LRTI deaths among newborns was mainly concentrated in low-SDI regions. Of the 204 countries or regions, 98 countries had mortality rates greater than 1,000 per 100,000 population (**Table 2**).

### Mortality of lower respiratory tract infections in all children aged 0–14 years from 1990 to 2021

**Figure 2:**
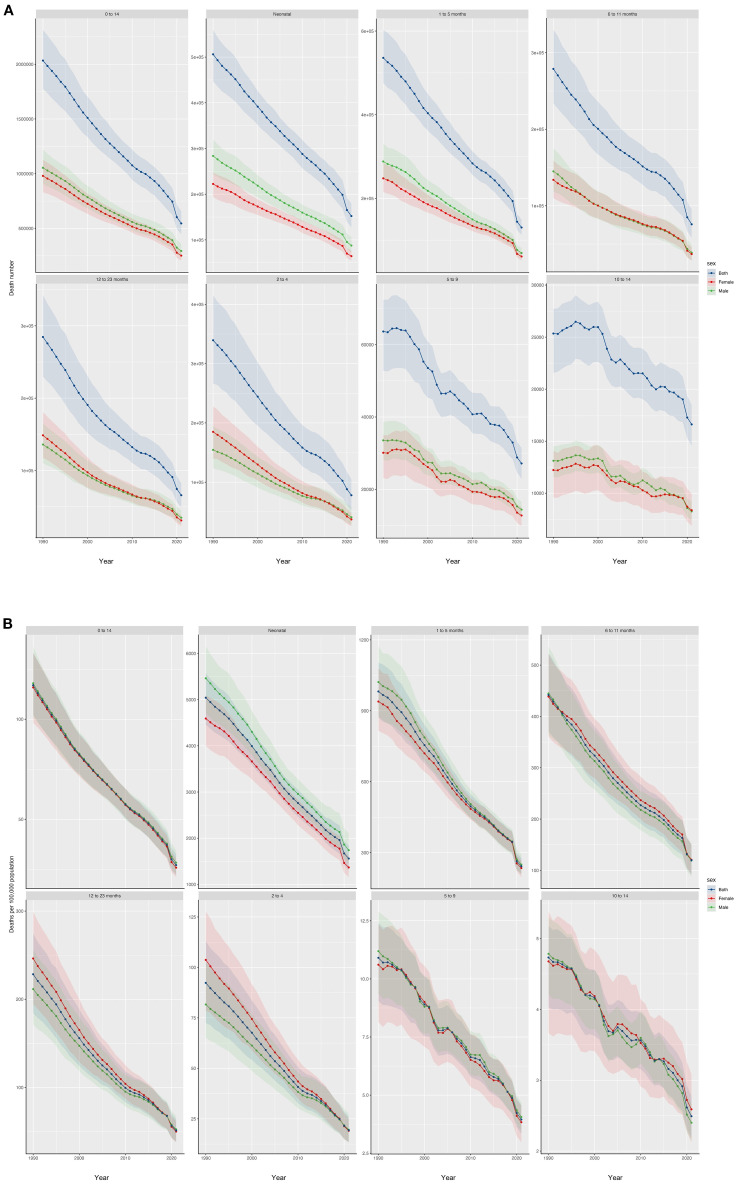
LRTI mortality rate and number of deaths from 1990 to 2021, stratified by age and sex The **(A)** number of deaths **(B)** mortality rate from 1990 to 2021, stratified by age and sex, are shown. The shaded areas represent 95% uncertainty intervals.

Globally, from 1990 to 2021, the all-age mortality rate of LRTIs among children aged 0–14 years decreased by 76.8% (95% UI 72.2–80.5), from 20.340 (95% UI 17.759 to 23.139) to 5.457 (95% UI 4.474 to 6.553) deaths per 100,000 population (**Figure 2**). Among boys, the mortality rate decreased by 76.0% (95% UI 71.0–80.1), from 10.539 (95% UI 9.069 to 12.189) to 2.941 (95% UI 2.372 to 3.573) deaths per 100,000 population (**Figure 2**). Among girls, the mortality rate decreased by 77.7% (95% UI 72.9–81.7), from 9.801 (95% UI 8.306 to 11.259) to 2.516 (95% UI 2.060 to 2.968) deaths per 100,000 population (**Figure 2**). Overall, the changes in LRTI mortality rates among children aged 0–14 years were not significantly different between sexes. The decline in the number of deaths was primarily driven by the reduction in LRTI mortality rates among children aged 1–4 years, decreasing by 77.5% (95% UI 71.8–82.0) among those aged 12–23 months and by 79.1% (95% UI 72.8–84.4) among those aged 2–4 years (**Figure 2**). Similar to the incidence rate, the smallest decline in LRTI mortality rate was observed among children aged 10–14 years (47.3%, 95% UI 40.6–52.8) (**Figure 2**).

### Risk factors for LRTIs in children aged 0–14 years from 1990 to 2021

**Figure 3:**
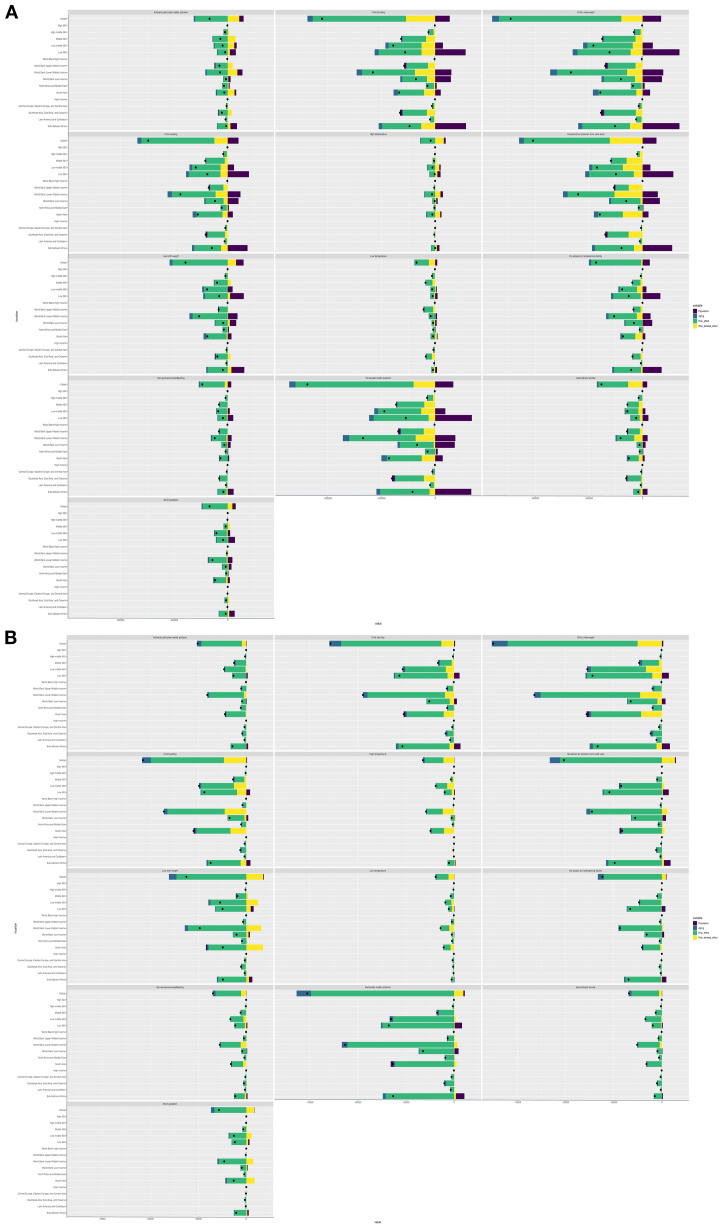
Risk factors for LRTIs in children aged 0–14 years in 1990–2019 and 2019–2021 analyses of risk factors for LRTIs in children aged 0–14 years **(A)** from 1990 to 2019 and **(B)** from 2019 to 2021.

In 2019, before the COVID-19 pandemic, the total number of DALYs attributable to LRTI risk factors was 59.746 million (95% UI 49.011 to 71.078 million), representing a reduction of 376.7% (95% UI 365.3 to 387.8), compared with 168.373 million (95% UI 144.370 to 194.828) in 1990. According to the GBD framework, the 13 risk factors for LRTI burden among children aged 0–14 years include underweight children, particulate matter pollution, childhood stunting, household air pollution from solid fuels, child wasting, low birth weight, lack of access to handwashing facilities, ambient particulate matter pollution, short gestation, secondhand smoke, nonexclusive breastfeeding, high temperature, and low temperature. The leading risk factor was low birth weight (2.627 million DALYs, 95% UI 1.065 to 4.342 million), followed by particulate matter pollution (2.610 million DALYs, 95% UI 0.750 to 4.251 million); child stunting (1.941 million DALYs, 95% UI 1.315 to 2.564 million); household air pollution from solid fuels (1.824 million DALYs, 95% UI 0.563 to 3.200 million; and child wasting (1.493 million DALYs, 95% UI 0.956 to 2.054 million). High and low temperatures were found to have minimal correlation with LRTI DALYs (**Figure 3**).

Compared with the data in 2019, the LRTI burden among children aged 0–14 years in 2021 remained attributable to the same 13 risk factors, although the number of DALYs decreased by 74.2% (95% UI 69.0 to 78.6) at 4.345 million (95% UI 3.434 to 5.247 million). However, the leading risk factor became particulate matter pollution, accounting for 1.930 million DALYs (95% UI 0.573 to 3.218 million) and with 75.6% reduction (95% UI 69.8 to 80.2). The second leading risk factor was child underweight (1.845 million DALYs, 95% UI 0.071 to 3.116 million), which decreased by 77.2% (95% UI 72.3 to 82.5). Household air pollution from solid fuels was the third leading risk factor, with 1.371 million DALYs (95% UI 0.427 to 2.411 million) and a 78.5% reduction (95% UI 72.4 to 83.3). The fourth and fifth leading risk factors were child stunting (1.369 million DALYs, 95% UI 0.926 to 1.830 million) and child wasting (1.013 million DALYs, 95% UI 0.631 to 1.440 million), respectively. Notably, from 2019 to 2021, the ranking of particulate matter pollution and household air pollution from solid fuels increased, whereas high and low temperatures remained minimally associated with LRTI DALYs among children aged 0–14 years (**Figure 3**).

### Analysis of the main pathogens and worldwide burden of lower respiratory tract infections among children aged 0–14 years in 2021

In 2021, influenza A/H3N2 was the dominant subtype globally, accounting for 58.3% (95% UI 52.1-64.5) of influenza-associated LRTI deaths in children aged 0-14 years, followed by influenza B (27.6%, 95% UI 22.4-33.1) and A/H1N1 (14.1%, 95% UI 10.3-18.7). The mortality burden was disproportionately concentrated in low-middle SDI regions, where influenza A/H3N2 caused 23.7 deaths per 100,000 (95% UI 19.2-28.9) - 3.2-fold higher than high-SDI regions. Vaccination coverage showed stark regional disparities: ≤15% in Sub-Saharan Africa vs. 63.2% in North America (WHO/UNICEF 2021 estimates). Notably, seasons with A/H3N2 dominance correlated with 42% higher mortality than H1N1-dominant years (p<0.001).

The top three pathogens contributing to LRTI-related DALYs among children aged 0–14 years globally were *S. pneumoniae* (135.877 DALYs per 100,000 population, 95% UI 108.004–163.224); *S. aureus* (47.620 DALYs per 100,000 population, 95% UI 38.402–57.603); and *K. pneumoniae* (436.123 million DALYs, 95% UI 347.301–532.681).

Among the high-, high–middle-, and middle-SDI regions, the top three pathogens contributing to LRTI-related DALYs and deaths in this age group were *S. pneumoniae*, *S. aureus*, and other viruses. In low–middle-SDI regions, the top three pathogens were *S. pneumoniae*, *S. aureus*, and *K. pneumoniae*. In high-SDI regions, *S. pneumoniae* contributed 0.233 DALYs (95% UI 0.211–0.255) and 0.003 deaths (95% UI 0.002–0.003) per 100,000 population. In low-SDI regions, *S. pneumoniae* contributed 67.745 DALYs (95% UI 50.154–85.595) and 0.769 deaths (95% UI 0.569–0.072) per 100,000 population. *S. aureus* contributed 0.213 DALYs (95% UI 0.195–0.228) and 0.002 deaths (95% UI 0.002–0.003) per 100,000 population in high-SDI regions and 29.036 DALYs (95% UI 17.539–29.036) and 26.334 deaths (95% UI 19.929–32.064) per 100,000 population in low-SDI regions The pattern was similar among the countries stratified by income.

**Figure 4:**
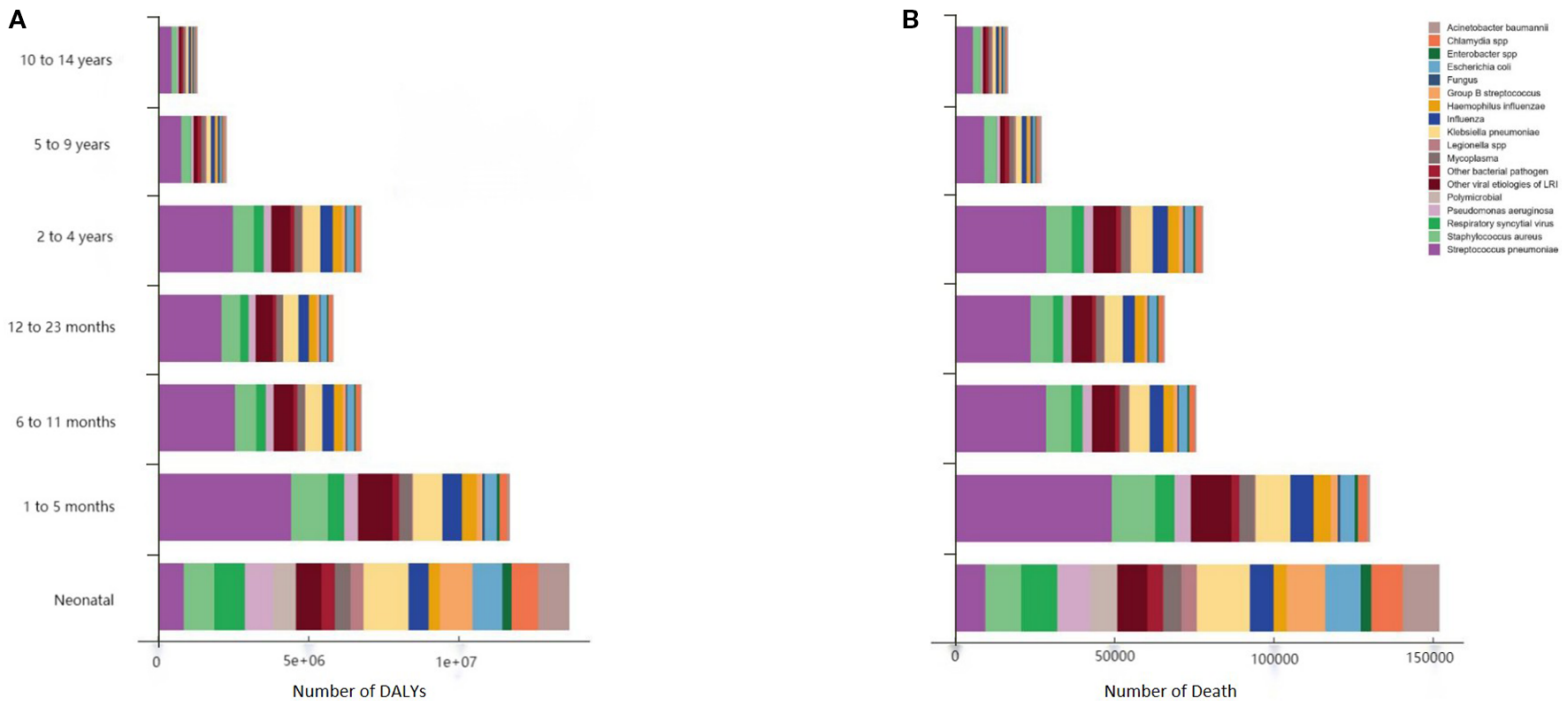
Etiological DALYs and number of deaths due to LRTIs among children globally in 2021, stratified by age group. The **(A)** DALYs and **(B)** number of deaths due to LRTIs; “Other viruses” included all studied viruses, except influenza and respiratory syncytial virus. DALYs, distribution of disability-adjusted life years; LRTI, lower respiratory tract infection.

Among children aged 0–14 years across the 204 modeled countries and regions in 2021, *S. pneumoniae* was the leading cause of DALYs in 171 countries or regions, whereas *S. aureus* was the leading cause of LRTI deaths in the remaining 33 countries or regions. Across the seven age subgroups, *S. pneumoniae* was the most frequent pathogen causing LRTIs (**Figure 4**).

When stratified by age group, the top three pathogens contributing to LRTI-related DALYs and deaths among newborns aged 0–28 days globally were *K. pneumoniae*, Group B Streptococcus, and *Acinetobacter baumannii*. *K. pneumoniae* contributed 14.979 DALYs (95% UI 11.574–19.021) and 0.166 deaths (95% UI 0.129–0.211) per 100,000 population. Group B Streptococcus contributed 10.897 DALYs (95% UI 8.462–13.733) and 0.121 deaths (95% UI 0.094–0.153) per 100,000 population. *A. baumannii* contributed 10.397 DALYs (95% UI 5.507–17.695) and 0.116 deaths (95% UI 0.061–0.197) per 100,000 population. For children aged 1–5 months, 6–11 months, 2–4 years, 5–9 years, and 10–14 years, the top three pathogens contributing to LRTI-related DALYs and deaths were *S. pneumoniae*, *S. aureus*, and *K. pneumoniae* (**Figure 4**).

### Changes in main pathogens and burden of lower respiratory tract infection in children from 2019 to 2021

**Figure 5:**
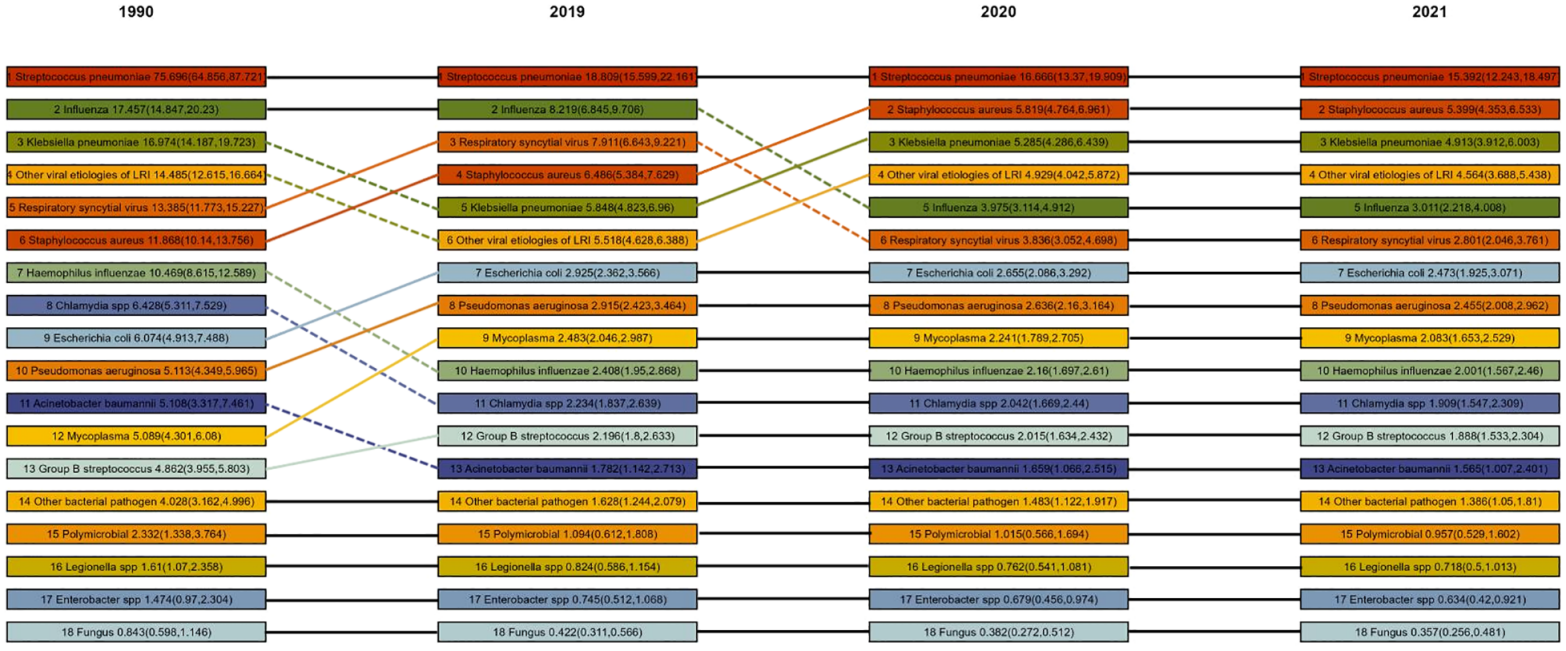
Global ranking of burden of death due to pathogens associated with lower respiratory tract infections among children aged 0–14 years in 1990, 2000, 2010, and 2021 Values represent the estimated number of deaths or DALYs per 100,000 population for each pathogen, with 95% uncertainty intervals shown in parentheses. Estimates are presented with three significant digits.

In 2019, during the COVID-19 pandemic, the top three pathogens causing LRTI deaths in children aged 0–14 years were the same as those in 1990: *S. pneumoniae*, influenza, and RSV. *S. pneumoniae* was the most common cause of LRTI deaths globally, resulting in 188,090 deaths (95% UI 155,990 to 221,610). Influenza was the second leading pathogen, causing 82,190 deaths (95% UI 68,450 to 97,060), and RSV was the third leading pathogen, causing 79,110 deaths (95% UI 66,430 to 92,210) (**Figure 5**). In 2019, *S. pneumoniae* was the leading cause of LRTI deaths in all age groups, except in newborns. The leading pathogen causing LRTI deaths in newborns was RSV (29,180 deaths, 95% UI 24,510 to 34,620), followed by *K. pneumoniae* (18,810 deaths, 95% UI 14,980 to 23,370) and influenza (18,400 deaths, 95% UI 14,420 to 23,120).

In 2021, the top three pathogens causing LRTI deaths in children aged 0–14 years were *S. pneumoniae* (153,920 deaths, 95% UI 122,430 to 184,970), followed by *S. aureus* (53,990 deaths, 95% UI 43,530 to 65,330) and *K. pneumoniae* (49,130 deaths, 95% UI 39,120 to 60,030). In 2021, *S. pneumoniae* remained the leading cause of LRTI deaths in all age groups, except in newborns (**Figure 4**). Among newborns, the leading cause of LRTI deaths was *K. pneumoniae* (16,650 deaths, 95% UI 12,860 to 21,140), followed by Group B Streptococcus (12,110 deaths, 95% UI 9,400 to 15,260) and *A. baumannii* (11,560 deaths, 95% UI 6,120 to 19,670). Unlike the top three pathogens causing LRTI deaths among children aged 0–14 years in 2021, other viral etiologies of LRTIs were the third leading cause of death among children under 5 years old, excluding newborns, causing 33,710 deaths (95% UI 26,330 to 41,440) (**Figure 4**). In 2021, the leading causes of LRTIS deaths across the 204 modeled countries and regions were *S. pneumoniae* in 172 countries and *S. aureus* in 31 countries. In Iceland, RSV was the leading cause of LRTI deaths in children aged 0–14 years.

From 1990 to 2019, the largest decrease in global mortality rate among children aged 0–14 years was from influenza (29.7% decrease, 95% UI 29.7–30.6), followed by Enterobacteriaceae (26.5% decrease, 95% UI 27.3–25.7). These improvements were most pronounced in children aged 2–4 years, with influenza-related deaths decreasing by 57.9% (95% UI 56.3–61.1), from 28,258.625 (95% UI 21,953.534–35,001.644) to 11,893.952 (95% UI 8,530.451–15,307.666), and Enterobacteriaceae-related deaths decreasing by 52.2% (95% UI 50.6–54.3), from 1,391.960 (95% UI 949.929–1,905.236) to 665.620 (95% UI 434.409–940.669).

After the start of the COVID-19 pandemic, we estimated that the number of influenza-related DALYs among children aged 0–14 years from 2019 to 2021 decreased by 63.4% (95% UI 52.1–73.0), from 7.279 million (95% UI 6.058–8.604) to 2.664 million (95% UI 1.962–3.547). A similar decrease of 63.4% (95% UI 52.0–72.9) was observed in the number of deaths, from 82,190 (95% UI 68,450–97,060) to 30,110 (95% UI 22,180–40,080). Among the 18 modeled pathogen categories, influenza decreased in rank from second in 2019 to fifth in 2021 as the leading cause of global LRTI DALYs and deaths among children aged 0–14 years (**Figure 5**). From 2019 to 2021, the mortality rate of influenza in this age group decreased the most in high-income regions (93.4%, 95% UI 89.6–96.0) and had the smallest decrease in Sub-Saharan Africa (57.3%, 95% UI 40.5–70.4).

Similarly, since 2019, the number of RSV-related DALYs globally has decreased by 64.6% (95% UI 53.8–74.2), from 7.069 million (95% UI 5.941–8.238) to 2.502 million (95% UI 1.828–3.358). Moreover, the number of RSV-related deaths decreased significantly by 64.6% (95% UI 53.7–74.2), from 79,110 (95% UI 66,430–92,210) in 2019 to 28,010 (95% UI 20,460–37,610) in 2021. Among the 18 modeled pathogen categories, RSV decreased in rank from third in 2019 to sixth in 2021 as the leading cause of global LRTI DALYs and deaths among children aged 0–14 years. Similar to influenza, the largest reduction in RSV mortality rates in this age group from 2019 to 2021 was observed in high-income regions (94.0%, 95% UI 90.7–96.3), whereas the smallest reduction was observed in Sub-Saharan Africa (57.6%, 95% UI 40.7–70.9).

Overall, for global non-COVID-19 LRTIs among children aged 0–14 years from 2019 to 2021, we estimated an 11.8% (95% UI 9.6–14.0) decrease in the overall incidence rate, from 790.176 (95% UI 706.289–897.168) to 699.005 (95% UI 621.350–797.806) per 100,000 population. The DALYs rate decreased by 27.1% (95% UI 22.3–31.2), from 483.630 (95% UI 396.663–580.345) to 661.162 (95% UI 554.592–773.710) per 100,000 population. The mortality rate decreased by 27.0% (95% UI 22.2–31.1), from 7.451 (95% UI 6.249–8.717) to 5.457 (95% UI 4.474–6.553) per 100,000 population.

### Prediction of mortality rates for 18 pathogens of LRTIs in children Aged 0–14 years globally from 2021 to 2031

Between 2021 and 2031, the mortality rate of LRTI in children showed a significant downward trend. In 2021, the mortality rate for LRI in children was 27.124 per 100,000 population (95% UI 26.930–27.317), but by 2031, this figure had sharply decreased to 9.032 per 100,000 population (95% UI 4.244–13.819).

Among the 18 pathogens studied, the mortality rate caused by the influenza virus exhibited a unique trend, with an increase over time, while the mortality rates associated with the other 17 pathogens declined to varying extents. Specifically, the mortality rate for children aged 0-14 years due to influenza virus in 2021 was 1.497 per 100,000 population (95% UI 1.993–1.102). This rate initially decreased to 1.071 per 100,000 population (95% UI 0– 21.976) in 2029 but then gradually rose to 4.482 per 100,000 population (95% UI 0–302.115) in 2031. The male mortality rate was approximately 2.906 per 100,000 population (95% UI 0–162.567), while the female mortality rate was approximately 6.160 per 100,000 population (95% UI 0 –450.896), making influenza virus the leading cause of death from LRI in children.

**Figure 6:**
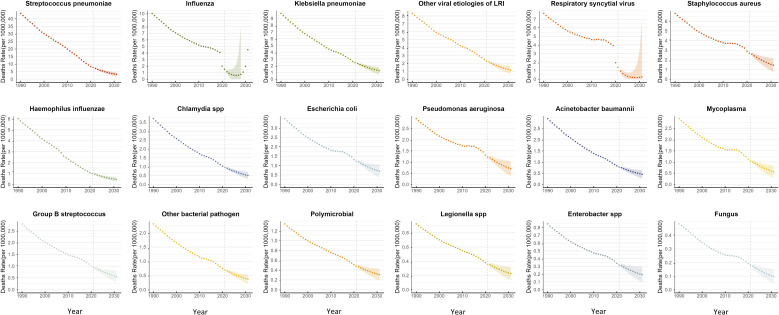
BAPC prediction of mortality rates for 18 pathogens of LRTIs in children aged 0-14 years globally from 2021 to 2031. The data from 1990 to 2021 are historical, while the BAPC predictions for 2021-2031 are presented with 95% UI.

In contrast, the mortality rate from pneumonia caused by Streptococcus pneumoniae for children aged 0-14 years declined from 7.651 per 100,000 population (95% UI 6.086–9.194) in 2021 to 3.129 per 100,000 population (95% UI 1.822–4.435) in 2031, with its ranking as the leading cause of death dropping to second place. Similarly, the mortality rate from Staphylococcus aureus also showed a notable decline, from 2.684 per 100,000 population (95% UI 2.163–3.247) in 2021 to 1.444 per 100,000 population (95% UI 0.720–2.167) in 2031, with its ranking as the second leading cause of death dropping to third place (**Figure 6**).

## Discussion

This study analyzed the incidence and mortality rates of non-COVID-19 LRTI from 1990 to 2050 among children aged 0–14 years at the global, regional, and national levels using data from the 2021 GBD study. The results indicated significant differences in pediatric LRTI burden among geographic regions over time, and these were related to multiple factors, such as major pathogens, risk factors, and SDI. This study on pediatric LRTIs focused on trends of global incidence among different age groups and the main pathogenic characteristics. The results showed that although the global incidence and mortality rates of pediatric LRTIs significantly declined over the past three decades, notable differences among specific regions and age groups remain.

The significant downward trend in the global incidence of LRTIs among children aged 0–14 years from 1990 to 2021 can be mainly attributed to the promotion of vaccination, improved public health measures, and widespread availability of healthcare resources (Antimicrobial Resistance, 2022). This significantly reduced incidence was particularly observed in middle- and high-income countries, which have implemented vaccination and enhanced medical care (Raj et al., 2595 2023). However, in low-income and low–middle-income countries, the incidence remains high. For instance, the highest incidence rates were observed among children in South Asia and Sub-Saharan Africa, reflecting inadequate medical conditions and health infrastructure in these regions (Collaborators GBDAR, 2024). Moreover, public health measures and lockdown policies during the pandemic likely altered pathogen transmission patterns to some extent, with significant declines in the rates of influenza virus and RSV infection (Tang et al., 2021). Studies have shown that although LRTI has a gradually decreasing overall incidence, its epidemiology was significantly affected by COVID-19-related public health measures. Isolation and preventive measures during the pandemic led to a temporary decline in LRTI incidence, especially in high-income countries; however, after 2021, LRTI cases have rebounded in number and present with more severe clinical phenotypes— a phenomenon known as immunity debt (Nagelkerke et al., 2024).

Neonates had the highest incidence of LRTIs, reaching 14,740.851 cases per 100,000 population in 2021. The incidence gradually decreased with age, dropping to 1,840.776 cases per 100,000 population among children aged 10–14 years. The high incidence in neonates was mainly due to underdeveloped immune systems, which likely increased the susceptibility to pathogen invasion (Feikin et al., 2024). Moreover, infants aged 1–5 months were found to be a high-risk group, reflecting the high susceptibility to respiratory infections among children in this age range.

Neonates and young children, especially those under 5 years old, were high-risk populations for LRTIs. During the COVID-19 pandemic, the significant decrease in LRTI incidence among infants under 1 year old was possibly related with social isolation measures and enhanced personal hygiene practices (Qiu et al., 2022). The varying incidence rates and LRTI pathogens among children of different age groups were likely due to differences in immune system development. Other studies have indicated that the COVID-19 pandemic not only altered the overall incidence trend of LRTIs but also changed the infection risk among children of different age groups, with younger children being more severely affected (Tang et al., 2021).

In 2021, *S. pneumoniae*, *S. aureus*, and *K. pneumoniae* were the main bacterial pathogens of pediatric LRTIs. Among them, *S. pneumoniae* was the leading cause of LRTI-related deaths globally, consistent with recent literature. Pneumococcal pneumonia caused 55.4% of all LRTI-related deaths across all age groups in high- and middle-income countries (Collaborators GL, 2017). Considering the relatively low vaccine coverage in low-income and low–middle-income countries, *S. pneumoniae* remains the primary pathogenic factor (Li et al., 2021).

Among older children, *S. pneumoniae*, *S. aureus*, and *K. pneumoniae* are relatively common pathogens (Raj et al., 2595 2023). Among older children (1 month to 9 years), *S. pneumoniae* and *S. aureus* were the bacterial pathogens significantly associated with severe pneumonia. These bacteria were reported to have relatively high prevalence rates in children with comorbidities (von Mollendorf et al., 2022), highlighting the need to implement targeted prevention and treatment strategies in this population. From 1990 to 2019, the significant decrease in *Haemophilus influenzae* and *S. pneumoniae* mortality rates by 77.4% and 76.1%, respectively, among children under 5 years old was mainly attributed to vaccination and improvements in medical conditions (Infections GBDLR and Antimicrobial Resistance C, 2024).

On the other hand, among neonates, this study found that the main pathogens were *K. pneumoniae*, Group B *Streptococcus*, and *A. baumannii*. *K. pneumoniae*, especially carbapenem-resistant and hypervirulent strains, poses a serious threat because of high mortality rates and treatment resistance. Group B *Streptococcus* is a leading cause of neonatal meningitis and sepsis and may have significant long-term impact on survivors. *A. baumannii* is a critical pathogen known for its antimicrobial resistance and association with severe infections (Broad et al., 2020; Furuta et al., 2022; Hu et al., 2023). These pathogens are relatively common in infants with weak immune systems. Eradicating these pathogens requires continuous surveillance, effective infection control measures, and development of new therapeutic strategies.

Despite widespread administration of pneumococcal conjugate and *H. influenzae* type b vaccines, bacterial pathogens remain important causes of moderate to severe disease in children with comorbidities (von Mollendorf et al., 2022). In addition, researchers have pointed out the urgent need to avoid overuse of antibiotics and reduce antimicrobial resistance in children with community-acquired pneumonia (Antimicrobial Resistance, 2022; Meyer Sauteur, 2024).

In 2019, the global leading cause of LRTI cases and deaths among children under 5 years old was *S. pneumoniae*, followed by RSV and influenza. In 2021, with the significant impact of the COVID-19 pandemic on influenza and RSV transmission, the incidence of influenza declined, but *S. pneumoniae* remained the most prevalent pathogen of LRTIs worldwide. Among children under 5 years old, RSV is the second most lethal pathogen after *S. pneumoniae (* Zdanowicz et al., 2023).

In this study, RSV was found to be the main virus causing neonatal death. In low-income and low–middle-income countries, RSV mainly affects younger children, highlighting the need for effective vaccines (Morgan et al., 2023). Over the past 30 years, the incidence rates of RSV and influenza have decreased; this trend was more pronounced in middle- and high-income countries, possibly because of better resources, management, and access to vaccines and monoclonal antibodies (Mezei et al., 2021). In low- and middle-income countries, prevention and management of RSV require improved diagnostics, education, timely access to new interventions, and engagement with policy makers (Carbonell-Estrany et al., 2022).

Our study have shown that the mortality rate from LRTIs has declined since the 1990s. However, despite a slight improvement in mortality rates from 18 LRTI pathogens over the next decade, influenza may cause an increase in childhood mortality starting in 2029, potentially becoming the leading cause of death among these pathogens, with higher mortality rates in females than in males. This trend is closely associated with the variability and immune escape properties of the influenza virus, as well as insufficient vaccination coverage, which result in some children’s immune systems being unable to effectively respond to new viral strains and a reduction in the protective efficacy of vaccines (Smyk et al., 2022; Han et al., 2023). Influenza leads to higher mortality rates by triggering excessive immune responses, causing secondary bacterial infections (e.g., pneumococcal infections), and inducing complications such as acute respiratory distress syndrome (ARDS) (Jugulete et al., 2024). The mortality rate from influenza is particularly high among high-risk populations, such as immunocompromised children and pregnant women. Additionally, gender differences in influenza infection outcomes may be attributed to variations in immune responses, with female children potentially facing higher mortality risks (Li L. et al., 2023). Given these factors, future public health strategies should be tailored to address susceptibility factors, with specific measures to mitigate the pathogenicity of various respiratory pathogens, especially influenza.

Our study identified particulate matter pollution, child malnutrition, and household solid fuel pollution as the primary risk factors contributing to LRTI burden in children. Underweight and malnutrition in children were also major risk factors leading to high LRTI incidence rates, similar with the research by Ruan et al (Ruan et al., 2021).

In China, the LRTI burden among children under 5 years old has significantly decreased, but it remains heavily influenced by particulate matter (PM) pollution, child malnutrition, and household solid fuel pollution (Li X. et al., 2023; Shi et al., 2023). The use of household solid fuels remains prevalent in low- and low–middle-income countries. Reducing exposure to environmental and household air pollutants and addressing child malnutrition are key strategies to mitigate the impact of LRTIs on global children’s health.

Our research found that in 2020 and 2021, the burden of pediatric LRTIs increased in low-income countries. This underscored the need for global public health to focus on the impact of the COVID-19 pandemic on children’s health. The COVID-19 pandemic disrupted routine childhood immunization programs, especially in low- and middle-income countries, leading to decreased vaccine coverage and making children more susceptible to LRTIs (Zar et al., 2020). The indirect pandemic effects, such as increased poverty and reduced access to healthcare, have further exacerbated this issue (Zar et al., 2020; Polasek et al., 2022). Despite these challenges, maintaining routine immunizations is crucial in order to prevent a large number of deaths and alleviate the burden of respiratory infections in children. Efforts should focus on sustaining and strengthening immunization programs to protect children’s health during and after the pandemic. After contracting COVID-19, children with chronic lung diseases, such as cystic fibrosis and asthma, are prone to more severe respiratory complications, such as acute respiratory distress syndrome and respiratory failure. For these patients, COVID-19 not only worsens the underlying condition but also increases the need for oxygen therapy and other intensive care treatments (Feldstein et al., 2020; Pardasani, 2022; Bembea et al., 2023). Therefore, there is a relatively high incidence of coexisting pulmonary infections that require more intensive medical interventions and monitoring (Kim et al., 2022).

Our prediction of rising influenza mortality is primarily driven by A/H3N2’s antigenic drift and low VE in pediatric populations. The 42% higher mortality in H3N2-dominant seasons underscores urgent needs for: (1) adjuvanted vaccines to improve cross-protection, (2) targeting ≥75% coverage in LMICs through GAVI partnerships, and (3) real-time strain surveillance in high-burden regions like South Asia where vaccine mismatch reached 68% in 2019-2021. The gender disparity in mortality (female:male RR=1.32 for A/H3N2) may reflect sex-dimorphic immune responses, warranting sex-stratified vaccine trials.

To reduce the future disease burden of pediatric LRTIs, our study recommends strengthening interventions in three areas. First, increase the coverage of pneumococcal vaccines, especially in low- and low–middle-income countries (Kim et al., 2022). Second, enhance control of air pollution, particularly particulate matter pollution and household solid fuel use. According to the research by Zubaidah Al-Janabi et al, improving household energy use and living conditions may effectively reduce the occurrence of pediatric LRTIs (Al-Janabi et al., 2021). Nevertheless, global public health interventions, including vaccination programs and policies that aim to improve air quality, have played a significant role in reducing the burden of LRTIs. In some countries, such as China, the incidence and mortality rates of pediatric LRTIs were significantly decreased by decreasing air pollution and promoting vaccination (Jin et al., 2021).Lastly, improve nutritional status, particularly by increasing aid to malnourished children, in order to significantly lower the incidence of LRTIs (Kirolos et al., 2021).

### Limitations

This study has several limitations. It relied on data from specific medical institutions and countries, which may limit the generalizability, especially in low- and middle-income countries with varying healthcare resources. Additionally, the Global Burden of Disease (GBD) data lacks detailed pathogen information, restricting the ability to fully analyze the LRTI burden. The study also considered comorbidities but did not explore specific conditions like chronic lung disease, asthma, and obesity, which may impact the severity of LRTIs. Furthermore, seasonal and regional variations were not analyzed, which are crucial for understanding differences in incidence and predominant pathogens. Future research should expand the sample size, incorporate seasonal and regional analyses, and include more detailed data on comorbidities to improve the understanding of pediatric LRTIs.

## Conclusions

This study analyzed the global trends and main pathogenic characteristics of pediatric LRTI. Although the incidence and mortality rates of LRTIs have declined globally, especially in high-income countries, the burden remains heavy in low- and middle-income countries. Moreover, younger children especially neonates were more susceptible to infections due to underdeveloped immune systems. *Streptococcus pneumoniae, Staphylococcus aureus, Klebsiella pneumoniae* were the most common causes of death in LRTIs. The burden of 18 common pathogens causing lower respiratory tract infections is expected to gradually decrease over the next decade, while the burden of influenza will steadily increase, becoming the leading cause of death, with higher mortality rates in females compared to males. The COVID-19 pandemic has reduced the transmission of respiratory viruses, but an ensuing immunity debt phenomenon led to more cases of severe infection, and the presence of COVID-19 is more likely to exacerbate underlying comorbidities among children. Particulate matter pollution and childhood underweight were the major risk factors contributing to the burden of LRTIs. Therefore, expanding vaccine coverage, improving sanitary conditions, and implementing early interventions for high-risk pediatric populations are crucial strategies to further reduce the burden of LRTIs.

## Data Availability

The original contributions presented in the study are included in the article/supplementary material. Further inquiries can be directed to the corresponding authors.

## References

[B1] Al-Janabi Z. Woolley K. E. Thomas G. N. Bartington S. E. (2021). A Cross-Sectional Analysis of the Association between Domestic Cooking Energy Source Type and Respiratory Infections among Children Aged under Five Years: Evidence from Demographic and Household Surveys in 37 Low-Middle Income Countries. Int. J. Environ. Res. Public Health 18, 8516. doi: 10.3390/ijerph18168516, PMID: 34444264 PMC8394069

[B2] Antimicrobial Resistance C. (2022). Global burden of bacterial antimicrobial resistance in 2019: a systematic analysis. Lancet. 399, 629–655. doi: 10.1016/S0140-6736(21)02724-0, PMID: 35065702 PMC8841637

[B3] Bembea M. M. Loftis L. L. Thiagarajan R. R. Young C. C. McCadden T. P. Newhams M. M.. (2023). Extracorporeal membrane oxygenation characteristics and outcomes in children and adolescents with COVID-19 or multisystem inflammatory syndrome admitted to U.S. ICUs. Pediatr. Crit. Care Med. 24, 356–371. doi: 10.1097/PCC.0000000000003212, PMID: 36995097 PMC10153593

[B4] Broad J. Le Doare K. Heath P. T. Hallchurch P. Whelan I. Boyd H.. (2020). The current state of immunization against Gram-negative bacteria in children: a review of the literature. Curr. Opin. Infect. Dis. 33, 517–529. doi: 10.1097/QCO.0000000000000687, PMID: 33044242

[B5] Carbonell-Estrany X. Simoes E. A. F. Bont L. J. Gentile A. Homaira N. Scotta M. C.. (2022). Identifying the research, advocacy, policy and implementation needs for the prevention and management of respiratory syncytial virus lower respiratory tract infection in low- and middle-income countries. Front. Pediatr. 10, 1033125. doi: 10.3389/fped.2022.1033125, PMID: 36440349 PMC9682277

[B6] Collaborators GBDAR (2024). Global burden of bacterial antimicrobial resistance 1990-2021: a systematic analysis with forecasts to 2050. Lancet. 404, 1199–1226. doi: 10.1016/S0140-6736(24)01867-1, PMID: 39299261 PMC11718157

[B7] Collaborators GL (2017). Estimates of the global, regional, and national morbidity, mortality, and aetiologies of lower respiratory tract infections in 195 countries: a systematic analysis for the Global Burden of Disease Study 2015. Lancet Infect. Dis. 17, 1133–1161. doi: 10.1016/S1473-3099(17)30396-1, PMID: 28843578 PMC5666185

[B8] Feikin D. R. Karron R. A. Saha S. K. Sparrow E. Srikantiah P. Weinberger D. M.. (2024). The full value of immunisation against respiratory syncytial virus for infants younger than 1 year: effects beyond prevention of acute respiratory illness. Lancet Infect. Dis. 24, e318–ee27. doi: 10.1016/S1473-3099(23)00568-6, PMID: 38000374

[B9] Feldstein L. R. Rose E. B. Horwitz S. M. Collins J. P. Newhams M. M. Son M. B. F.. (2020). Multisystem inflammatory syndrome in U.S. Children and adolescents. New Engl. J. Med. 383, 334–346. doi: 10.1056/NEJMoa2021680, PMID: 32598831 PMC7346765

[B10] Furuta A. Brokaw A. Manuel G. Dacanay M. Marcell L. Seepersaud R.. (2022). Bacterial and host determinants of group B streptococcal infection of the neonate and infant. Front. Microbiol. 13, 820365. doi: 10.3389/fmicb.2022.820365, PMID: 35265059 PMC8899651

[B11] Han A. X. de Jong S. P. J. Russell C. A. (2023). Co-evolution of immunity and seasonal influenza viruses. Nat. Rev. Microbiol. 21, 805–817. doi: 10.1038/s41579-023-00945-8, PMID: 37532870

[B12] Hu Y. Yang Y. Feng Y. Fang Q. Wang C. Zhao F.. (2023). Prevalence and clonal diversity of carbapenem-resistant Klebsiella pneumoniae causing neonatal infections: A systematic review of 128 articles across 30 countries. PloS Med. 20, e1004233. doi: 10.1371/journal.pmed.1004233, PMID: 37339120 PMC10281588

[B13] Infections GBDLR Antimicrobial Resistance C (2024). Global, regional, and national incidence and mortality burden of non-COVID-19 lower respiratory infections and aetiologies, 1990-2021: a systematic analysis from the Global Burden of Disease Study 2021. Lancet Infect. Dis. 24, 974–1002. doi: 10.1016/S1473-3099(24)00176-2, PMID: 38636536 PMC11339187

[B14] Jia R. Lu L. Su L. Lin Z. Gao D. Lv H.. (2022). Resurgence of respiratory syncytial virus infection during COVID-19 pandemic among children in Shanghai, China. Front. Microbiol. 13, 938372. doi: 10.3389/fmicb.2022.938372, PMID: 35875547 PMC9298468

[B15] Jin W. Huang K. Ding Z. Zhang M. Li C. Yuan Z.. (2025). Global, regional, and national burden of esophageal cancer: a systematic analysis of the Global Burden of Disease Study 2021. biomark. Res. 13, 3. doi: 10.1186/s40364-024-00718-2, PMID: 39762900 PMC11702276

[B16] Jin X. Ren J. Li R. Gao Y. Zhang H. Li J.. (2021). Global burden of upper respiratory infections in 204 countries and territories, from 1990 to 2019. EClinicalMedicine. 37, 100986. doi: 10.1016/j.eclinm.2021.100986, PMID: 34386754 PMC8343248

[B17] Jugulete G. Olariu M. C. Stanescu R. Luminos M. L. Pacurar D. Pavelescu C.. (2024). The Clinical Effectiveness and Tolerability of Oseltamivir in Unvaccinated Pediatric Influenza Patients during Two Influenza Seasons after the COVID-19 Pandemic: The Impact of Comorbidities on Hospitalization for Influenza in Children. Viruses 16, 1576. doi: 10.3390/v16101576, PMID: 39459910 PMC11512198

[B18] Kim S. H. Hong J. Y. Bae S. Lee H. Wi Y. M. Ko J. H.. (2022). Risk factors for coronavirus disease 2019 (COVID-19)-associated pulmonary aspergillosis in critically ill patients: A nationwide, multicenter, retrospective cohort study. J. Korean Med. Sci. 37, e134. doi: 10.3346/jkms.2022.37.e134, PMID: 35535369 PMC9091428

[B19] Kirolos A. Blacow R. M. Parajuli A. Welton N. J. Khanna A. Allen S. J.. (2021). The impact of childhood malnutrition on mortality from pneumonia: a systematic review and network meta-analysis. BMJ Glob Health 6, e007411. doi: 10.1136/bmjgh-2021-007411, PMID: 34848440 PMC8634228

[B20] Li L. Yan Z. L. Luo L. Liu W. Yang Z. Shi C.. (2023). Influenza-associated excess mortality by age, sex, and subtype/lineage: population-based time-series study with a distributed-lag nonlinear model. JMIR Public Health Surveill. 9, e42530. doi: 10.2196/42530, PMID: 36630176 PMC9878364

[B21] Li X. Li Y. Yu B. Nima Q. Meng H. Shen M.. (2023). Urban-rural differences in the association between long-term exposure to ambient particulate matter (PM) and malnutrition status among children under five years old: A cross-sectional study in China. J. Glob Health 13, 04112. doi: 10.7189/jogh.13.04112, PMID: 37736866 PMC10515095

[B22] Li X. Mukandavire C. Cucunuba Z. M. Echeverria Londono S. Abbas K. Clapham H. E.. (2021). Estimating the health impact of vaccination against ten pathogens in 98 low-income and middle-income countries from 2000 to 2030: a modelling study. Lancet. 397, 398–408. doi: 10.1016/S0140-6736(20)32657-X, PMID: 33516338 PMC7846814

[B23] Meyer Sauteur P. M. (2024). Childhood community-acquired pneumonia. Eur. J. Pediatr. 183, 1129–1136. doi: 10.1007/s00431-023-05366-6, PMID: 38112800 PMC10950989

[B24] Mezei A. Cohen J. Renwick M. J. Atwell J. Portnoy A. (2021). Mathematical modelling of respiratory syncytial virus (RSV) in low- and middle-income countries: A systematic review. Epidemics. 35, 100444. doi: 10.1016/j.epidem.2021.100444, PMID: 33662812 PMC8262087

[B25] Morgan N. Buys H. Muloiwa R. (2023). RSV infection in children hospitalised with severe lower respiratory tract infection in a low-middle-income setting: A cross-sectional observational study. PloS One 18, e0291433. doi: 10.1371/journal.pone.0291433, PMID: 37708173 PMC10501652

[B26] Mrcela D. Markic J. Zhao C. Viskovic D. V. Milic P. Copac R.. (2022). Changes following the Onset of the COVID-19 Pandemic in the Burden of Hospitalization for Respiratory Syncytial Virus Acute Lower Respiratory Infection in Children under Two Years: A Retrospective Study from Croatia. Viruses 14, 2746. doi: 10.3390/v14122746, PMID: 36560751 PMC9785187

[B27] Nagelkerke M. C. M. Zagten M. V. Sprij A. Bekhof J. Veen M. V. Kruizinga M. D. (2024). Incidence, severity, and clinical characteristics of lower respiratory tract infections in children before and after the coronavirus disease 2019 lockdown: a Dutch single-center study. Pediatr. Emerg. Med. J. 11, 17–27. doi: 10.22470/pemj.2023.00836

[B28] Pardasani S. A. (2022). 44 COVID-19 among pediatric patients with pre-existing pulmonary conditions: Preliminary results from the Pediatric COVID-19 U.S. Registry. J. Pediatr. Infect. Dis. Soc. 11, S7–SS. doi: 10.1093/jpids/piac041.026

[B29] Polasek O. Wazny K. Adeloye D. Song P. Chan K. Y. Bojude D. A.. (2022). Research priorities to reduce the impact of COVID-19 in low- and middle-income countries. J. Glob Health 12, 09003. doi: 10.7189/jogh.12.09003, PMID: 35475006 PMC9010705

[B30] Qiu W. Zheng C. Huang S. Zhang Y. Chen Z. (2022). Epidemiological trend of RSV infection before and during COVID-19 pandemic: A three-year consecutive study in China. Infection Drug resistance. 15, 6829–6837. doi: 10.2147/IDR.S388231, PMID: 36465809 PMC9717604

[B31] Raj D. Rao Mallyala S. Chirumamilla A. Gajjar J. Patel S. Dekhne A.. (2023). 2595. Burden of lower respiratory tract infection in United States of america and its trend from 1990-2019: A benchmarking analysis from the global burden of disease study. Open Forum Infect. Dis. 10, ofad500.2210. doi: 10.1093/ofid/ofad500.2210

[B32] Ruan Z. Qi J. Qian Z. M. Zhou M. Yang Y. Zhang S.. (2021). Disease burden and attributable risk factors of respiratory infections in China from 1990 to 2019. Lancet Reg. Health West Pac. 11, 100153. doi: 10.1016/j.lanwpc.2021.100153, PMID: 34327361 PMC8315661

[B33] Safiri S. Mahmoodpoor A. Kolahi A. A. Nejadghaderi S. A. Sullman M. J. M. Mansournia M. A.. (2022). Global burden of lower respiratory infections during the last three decades. Front. Public Health 10, 1028525. doi: 10.3389/fpubh.2022.1028525, PMID: 36699876 PMC9869262

[B34] Sah R. Zaman K. Mohanty A. Al-Ahdal T. Awad H. Padhi B. K.. (2023). Respiratory syncytial virus with ongoing COVID-19: is it an emerging threat? Ann. Med. Surg. (Lond) 85, 67–70. doi: 10.1097/MS9.0000000000000153, PMID: 36742116 PMC9893426

[B35] Shi X. Wu M. Jia X. Bao J. Wang Y. Yang C.. (2023). Trends of Incidence, Mortality, and Risk Factors for Lower Respiratory Infections among Children under 5 Years in China from 2000 to 2019. Int. J. Environ. Res. Public Health 20, 3547. doi: 10.3390/ijerph20043547, PMID: 36834242 PMC9965335

[B36] Shin Y. H. Hwang J. Kwon R. Lee S. W. Kim M. S. Collaborators GBDAD. (2023). Global, regional, and national burden of allergic disorders and their risk factors in 204 countries and territories, from 1990 to 2019: A systematic analysis for the Global Burden of Disease Study 2019. Allergy. 78, 2232–2254. doi: 10.1111/all.15807, PMID: 37431853 PMC10529296

[B37] Smyk J. M. Szydlowska N. Szulc W. Majewska A. (2022). Evolution of influenza viruses-drug resistance, treatment options, and prospects. Int. J. Mol. Sci. 23, 12244. doi: 10.3390/ijms232012244, PMID: 36293099 PMC9602850

[B38] Tang X. Dai G. Jiang X. Wang T. Sun H. Chen Z.. (2021). Clinical characteristics of pediatric respiratory tract infection and respiratory pathogen isolation during the coronavirus disease 2019 pandemic. Front. Pediatr. 9, 759213. doi: 10.3389/fped.2021.759213, PMID: 35071128 PMC8767000

[B39] von Mollendorf C. Berger D. Gwee A. Duke T. Graham S. M. Russell F. M.. (2022). Aetiology of childhood pneumonia in low- and middle-income countries in the era of vaccination: a systematic review. J. Glob Health 12, 10009. doi: 10.7189/jogh.12.10009, PMID: 35866332 PMC9305023

[B40] Wang X. Li Y. Mei X. Bushe E. Campbell H. Nair H. (2021). Global hospital admissions and in-hospital mortality associated with all-cause and virus-specific acute lower respiratory infections in children and adolescents aged 5-19 years between 1995 and 2019: a systematic review and modelling study. BMJ Glob Health 6, e006014. doi: 10.1136/bmjgh-2021-006014, PMID: 34261758 PMC8281096

[B41] Wu X. Lu W. Sang X. Xu Y. Wang T. Zhan X.. (2023). Timing of bronchoscopy and application of scoring tools in children with severe pneumonia. Ital J. Pediatr. 49, 44. doi: 10.1186/s13052-023-01446-3, PMID: 37024936 PMC10079491

[B42] Yigezu A. Misganaw A. Getnet F. Berheto T. M. Walker A. Zergaw A.. (2023). Burden of lower respiratory infections and associated risk factors across regions in Ethiopia: a subnational analysis of the Global Burden of Diseases 2019 study. BMJ Open 13, e068498. doi: 10.1136/bmjopen-2022-068498, PMID: 37666561 PMC10481843

[B43] Zar H. J. Dawa J. Fischer G. B. Castro-Rodriguez J. A. (2020). Challenges of COVID-19 in children in low- and middle-income countries. Paediatr. Respir. Rev. 35, 70–74. doi: 10.1016/j.prrv.2020.06.016, PMID: 32654854 PMC7316049

[B44] Zdanowicz K. Lewandowski D. Majewski P. Polkosnik K. Liwoch-Nienartowicz N. Reszec-Gielazyn J.. (2023). Clinical presentation and co-detection of respiratory pathogens in children under 5 years with non-COVID-19 bacterial and viral respiratory tract infections: A prospective study in Bialystok, Poland (2021-2022). Med. Sci. monitor: Int. Med. J. Exp. Clin. Res. 29, e941785. doi: 10.12659/MSM.941785, PMID: 37794657 PMC10563589

[B45] Zhu X. Ye T. Zhong H. Luo Y. Xu J. Zhang Q.. (2022). Distribution and drug resistance of bacterial pathogens associated with lower respiratory tract infection in children and the effect of COVID-19 on the distribution of pathogens. Can. J. Infect. Dis. Med. Microbiol. 2022, 1181283. doi: 10.1155/2022/1181283, PMID: 35368516 PMC8965734

